# Preparation of Nb^5+^ Doped Na_3_V_2_(PO_4_)_3_ Cathode Material for Sodium Ion Batteries

**DOI:** 10.3390/ma17112697

**Published:** 2024-06-03

**Authors:** Jingming Wan, Xu Yang, Tian Xia

**Affiliations:** 1 Department of Chemistry, Zhejiang University, Hangzhou 310058, China; 2 College of Materials and Chemistry & Chemical Engineering, Chengdu University of Technology, Chengdu 610059, China; yangxu_2024@163.com (X.Y.); xiatian_work@163.com (T.X.)

**Keywords:** Na_3_V_2_(PO_4_)_3_, sodium-ion battery, Nb, cathode

## Abstract

Sodium-ion batteries (SIBs) have emerged as a promising alternative to lithium-ion batteries (LIBs) due to the abundance and low cost of sodium resources. Cathode material plays a crucial role in the performance of sodium ion batteries determining the capacity, cycling stability, and rate capability. Na_3_V_2_(PO_4_)_3_ (NVP) is a promising cathode material due to its stable three-dimensional NASICON structure, but its discharge capacity is low and its decay is serious with the increase of cycle period. We focused on modifying NVP cathode material by coating carbon and doping Nb^5+^ ions for synergistic electrochemical properties of carbon-coated NVP@C as a cathode material. X-ray diffraction analysis was performed to confirm the phase purity and crystal structure of the Nb^5+^ doped NVP material, which exhibited characteristic diffraction peaks that matched well with the NASICON structure. Nb^5+^-doped NVP@C@Nb_x_ materials were prepared using the sol–gel method and characterized by X-ray Diffraction (XRD), Scanning Electron Microscopy (SEM), Raman and Brunauer -Emmett-Teller (BET) analysis. First-principles calculations were performed based on density functional theory. VASP and PAW methods were chosen for these calculations. GGA in the PBE framework served as the exchange-correlation functional. The results showed the NVP unit cell consisted of six NVP structural motifs, each containing octahedral VO_6_ and tetrahedral PO_4_ groups to form a polyanionomer [V_2_(PO_4_)_3_] along with the c-axis direction by PO_4_ groups, which had Na1(6b) and Na2(18e) sites. And PDOS revealed that after Nb doping, the d orbitals of the Nb atoms also contributed electrons that were concentrated near the Fermi surface. Additionally, the decrease in the effective mass after Nb doping indicated that the electrons could move more freely through the material, implying an enhancement of the electron mobility. The electrochemical properties of the Nb^5+^ doped NVP@C@Nb cathode material were evaluated through cyclic voltammetry (CV), galvanostatic charge-discharge tests, electrochemical impedance spectroscopy (EIS), and X-ray photoelectric spectroscopy (XPS). The results showed that NVP@C@Nb_0.15_ achieved an initial discharge capacity as high as 114.27 mAhg^−1^, with a discharge capacity of 106.38 mAhg^−1^ maintained after 500 cycles at 0.5C, and the retention rate of the NVP@C@Nb_0.15_ composite reached an impressive 90.22%. NVP@C@Nb_0.15_ exhibited low resistance and high capacity, enabling it to create more vacancies and modulate crystal structure, ultimately enhancing the electrochemical properties of NVP. The outstanding performance can be attributed to the Nb^5+^-doped carbon layer, which not only improves electronic conductivity but also shortens the diffusion length of Na^+^ ions and electrons, as well as reduces volume changes in electrode materials. These preliminary results suggested that the as-obtained NVP@C@Nb_0.15_ composite was a promising novel cathode electrode material for efficient sodium energy storage.

## 1. Introduction

Rechargeable battery technology is a huge driving force for the energy storage and conversion industry, and it is a reliable, portable, and economically favorable energy storage technology for various applications [1]. Among various storage systems, LIBs have become the dominant choice in the electronic industry due to their high energy density and long cycle life; however, a scarcity of lithium resources and safety concerns of LIBs prompted researchers to seek cheaper and more secure alternative energy storage systems [2]. SIBs are widely considered to be an ideal alternative to LIBs because of their superior safety performance, excellent high, cost-effective production, and low-temperature performance for wide applications, such as portable electronic devices [3,4,5,6,7,8]. However, one major challenge facing SIBs is their relatively low energy and power density, which can significantly hinder their future commercial viability [9]. One way to address this challenge is by developing new materials or technologies that can improve the energy and power density of SIBs. For example, researchers have been exploring the use of graphene or other cathode to increase the surface area and conductivity of lithium-sulfur batteries, which could lead to higher performance and longer lifetime [10]. Another approach is to optimize the design of the battery cell itself, such as by using a more efficient structure or incorporating additional components that can help regulate the flow of ions. This can help increase the capacity and stability of the battery, making it more suitable for commercial applications [11]. Wang et al. [12] assembled an NVP@C all-solid-state battery, which had a discharge capacity of 75.7 mAh/g and a capacity retention rate of 81.97% after 100 cycles. Zhao et al. [13] synthesized Ti-doped Na_3_V_1.9_Ti_0.1_(PO_4_)_3_@C cathode materials with 69.5% capacity retention after 14,000 cycles.

The development of high-performance and long-life cathode electrode materials of SIBs has become a research hotspot and a major challenge [11,14]. The process of selecting and preparing cathode materials demands careful consideration. Firstly, high output voltage is a crucial requirement [15]. Additionally, the crystal structure of the material should be optimized to maximize its ability to accept Na^+^ ions [16], which is beneficial to increase the number of sites available for ion diffusion through various channels such as layer gaps, octahedral gaps, and tetrahedral gaps [2,17,18]. Presently, a variety of cathode materials, such as transition metal oxides, Prussian blue-like compounds, polyanion compounds, and other materials, exhibit extensive potential applications [19]. Yamada et al. successfully obtained disordered and ordered Na_2_RuO_3_ cathode materials by regulating the synthesis route. Ordered Na_2_RuO_3_ exhibited a specific capacity of up to 180 mAhg^−1^, whereas disordered Na_2_RuO_3_ only had a capacity of 130 mAhg^−1^. Prussian blue-like compounds with an open frame structure, abundant redox active sites [20,21], excellent structural stability, large ion channels, and lattice gaps are highly effective Na^+^ storage materials. However, due to the influence of lattice defects and crystalline water, their coulombic efficiency is low, resulting in poor cycle stability and structural stability. Wang et al. [22] employed a citrate-assisted controlled crystallization coprecipitation method to synthesize Ni-doped NaxNi_0.23_Fe_0.77_HCN. The specific capacity could reach 105.9 mAhg^−1^ under 0.2 Ag^−1^ current density. In contrast, polyanion compounds had higher structural stability, thermal stability, and rapid ion diffusion kinetics, which contribute to improving the cycle stability and safety of the battery. Duan et al. [23] synthesized the core-shell nanocomposite NVP@C using hydrothermal-assisted sol–gel method. After 700 cycles, the capacity retention of the core-shell nanocomposite NVP@C could reach 96.1% with an initial capacity of 104.3 mAhg^−1^ and 94.9 mAhg^−1^ at 0.5C and 5C, respectively. While transition metal oxides boast high specific capacity and simple synthesis methods, they also face limitations such as low energy density (less than 3.5 V), poor cycle stability, and high moisture sensitivity.

Polyanionic compounds have developed significant growth as positive electrode materials [24]. Among them, NASICON-structured polyanion-type cathodes [25,26] with the general formula Na_x_MPO_4_ (where M represents Fe, V, Mn, or Co) have garnered attention for their outstanding physical and chemical properties, making them suitable for large-scale energy storage [27,28]. Phosphate compounds, a typical representative of polyanionic compounds, include well-known NVP cathodes with a three-dimensional framework [29,30]. NASICON structural backbone forms a stable sodium accommodation site, and the open three-dimensional ion transport channel is conducive to the rapid intercalation/deintercalation of Na ions. NVPs possess a relatively high voltage platform (3.4 V), making them a potential candidate for use in SIBs [31]. However, the low electronic conductivity inherent in the NVP crystal structure leads to poor magnification and cycling properties [32]. Hu et al. [5] reported on the performance of carbon-coated sodium vanadium phosphate cathode materials during charge–discharge tests. The output voltage was about 3.4 V, and the reversible specific capacity was about 100 mAhg^−1^. After 80 cycles of charge–discharge, the capacity retained approximately 93% of its initial value, with a volume change of approximately 8.3% throughout the entire process. After carbon coating, a layered structure of graphitized carbon is coated on the surface of NVP. The covalent bonds are formed between one carbon atom and NVP, which free electrons may not move freely without an external electric field, but when an electric field is applied, they are driven to migrate and thus conduct electricity. To enhance the conductivity and capacity of NVP, a combination of ion doping and carbon coating techniques was employed to boost the electron conductivity of the electrode material.

The doping of metal atoms, nitrogen (N), phosphorus (P), boron (B), and sulfur (S) heteroatoms into NVP@C has become increasingly popular [33], which can alter the electron distribution of carbon atoms, thereby enhancing the conductivity of carbon materials, reducing the energy barrier for ion penetration, and ultimately improving the comprehensive performance of batteries. Huang et al. [34] ingeniously combined the in-situ carbon coating and nitrogen coating carbon doping techniques to construct a material with double carbon source coating (NVP@C@NC) on its surface. To date, the bulk doping of NVP includes the partial doping of V, (PO_4_)^3−^ and Na active sites. Liu et al. [35] developed an NVP@C cathode material doped with iron (Na_3_V_1.85_Fe_0.15_(PO_4_)_3_@C), which exhibited a capacity of 103.69 mAhg^−1^ at 1.0 C, stable cycling for 1000 times at 5 C, and a specific capacity exceeding 90 mAhg^−1^. This outstanding performance highlighted the effectiveness of the doping and coating techniques in enhancing the overall performance of NVP-based battery materials. To delve into the electrochemical properties, the influence on electrochemical properties of Nb^5+^doping and carbon coating was investigated. The study aimed to explore the homogeneous incorporation of Nb^5+^ into V-sites in the NVP systems, providing insight into the effect of V-site replacement by Nb^5+^ on its microstructure and electrical properties.

## 2. Materials and Methods

### 2.1. Material Preparation

The porous NVP was obtained through the sol–gel method combined with high temperature sintering treatment. NVP was obtained through a sol–gel method with a stoichiometric ratio of NH_4_VO_3_ (1.990 g, LookChem, Chengdu, China), Na_2_CO_3_ (1.670 g, LookChem, Chengdu), NH_4_H_2_PO_4_ (2.300 g, LookChem, Chengdu), glucose (7.500 g, LookChem, Chengdu), and Nb_2_O_5_ (0.797 g, LookChem, Chengdu), which were respectively dissolved in 80 mL of deionized water, resulting in a homogeneous solution. This solution was vigorously stirred at 35 °C for 2 h, followed by heating to 80 °C to evaporate the gel to obtain the pre-NVP. Next, the pre-NVP obtained was heated under an N_2_ atmosphere at 350 °C for 4 h and then at 800 °C for 8 h, resulting in the formation of Nb-dopedNa_3_V_2_(PO_4_)_3_, abbreviated as NVP@C@Nbx, (@C meant carbon coating, x was the proportion of Nb added to the raw material). The heating rate was 5 °C/min, and the N_2_ was 80 mL/min. The reactants were analytically pure without any further purifications.

### 2.2. Material Characterization

X-ray diffraction (XRD, UItima IV, Tokyo, Japan) was carried out to probe the phase structures of as-prepared materials. Raman spectra were collected with a 532 nm laser (LabRAM HR800, Horiba, Paris, France) to confirm the presence of graphene. The morphologies of the materials were characterized by scanning electron microscopy (SEM, Inspect F50, Thermo Fisher Scientific, Waltham, MA, USA). X-ray photoelectron spectroscopy (XPS, Escalab 250Xi, Thermo Fisher Scientific, MA, USA) was used to elucidate the elemental states in the materials. The specific surface area was determined by The Brunauer−Emmett−Teller (BET, ASAP 2460, Micromeritics, Norcross, GA, USA) method.

First-principles calculations were performed based on density functional theory. The Vienna ab initio simulation package (VASP), implementing the projector augmented wave (PAW) method, was chosen for these calculations. The general gradient approximation (GGA) in the Perdew–Burke–Ernzerhof (PBE) framework served as the exchange-correlation functional. A plane-wave basis set with a cutoff energy of 400 eV was employed in the computational process. A plane-wave basis set with a cutoff energy of 400 eV was used in the calculation, which can ensure that the total energy converges to 1 meV/atom. The Brillouin zone (BZ) was sampled with a mesh of 4 × 4 × 2 k-points for bulk crystal. All atoms were optimized until the maximum absolute forces of all atoms converged to 0.05 eV/Å.

### 2.3. Electrochemical Measurements

The electrochemical performances of the full-cell were evaluated by self-assembling CR-2016 cells. The working electrode was composed of the active material, which was mixed with 10% Super P and 10% polyvinylidene fluoride (PVDF), dissolved in N-methyl-2-pyrrolidone (NMP), and subsequently coated on Al foil. This mixture was dried at 80 °C for 12 h to achieve a desired electrode mass loading of approximately 1 mg cm^−2^. In the Na-ion full-cell setup, NVP@C@Nb composite coated onto aluminum foil was utilized as the anode material, while Na metal served as the cathode. The electrolyte employed was a 1 M NaClO_4_ solution in propylene carbonate (PC) containing 5% fluoroethylene carbonate (FEC), and a glass fiber separator was used to maintain the electrolyte integrity. An excess amount was used to ensure sufficient active material mass for the anode, and the voltage window for the full cell was set to 2.4–4.2 V. Then, cyclic voltammetry (CV) and electrochemical impedance spectroscopy (EIS) were conducted using a PGSTAT-30 electrochemical workstation. Galvanostatic charge/discharge tests were carried out using a LAND measurement system to provide further insights into the electrochemical behavior and cycle life of the full cell.

## 3. Results and Discussion

**Figure 1:**
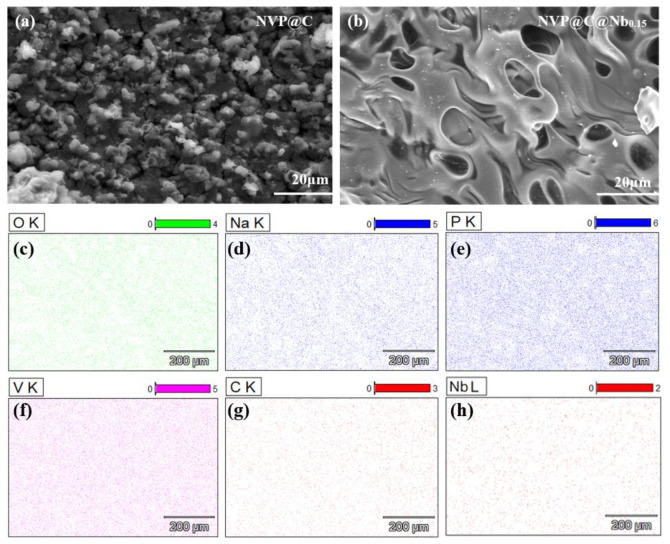
(**a**,**b**) SEM images of NVP@C and NVP@C@Nb_0.15_. (**c**–**h**) EDX plot of NVP@C@Nb_0.15_.

The surface morphology of all samples was investigated through SEM, and the results showed that the undoped NVP@C exhibited an agglomerated morphology with a rough surface (Figure 1a). In contrast, Nb-doped NVP@C@Nbx materials presented a comparatively smoother surface with distinct pores, approximately 10 nm, indicating a loose and porous structure (Figure 1b). Energy Dispersive X-ray (EDX) mapping analysis demonstrated the uniform distribution of all constituent elements, including C, Na, V, P, O, and Nb, across the materials (Figure 1c–h). These results clearly demonstrated the successful accomplishment of simultaneous structural modification through Nb substitution and surface carbonation of the NVP cathode material. The partial incorporation of Nb^5+^ ions hindered the growth of NVP@C, as these ions occupied the lattice sites. Consequently, this increased the number of lattice defects and provided an abundance of active sites. And the presence of numerous pores within the samples could facilitate the creation of Na^+^ transport channels to enhance the electrochemical performances.

**Figure 2:**
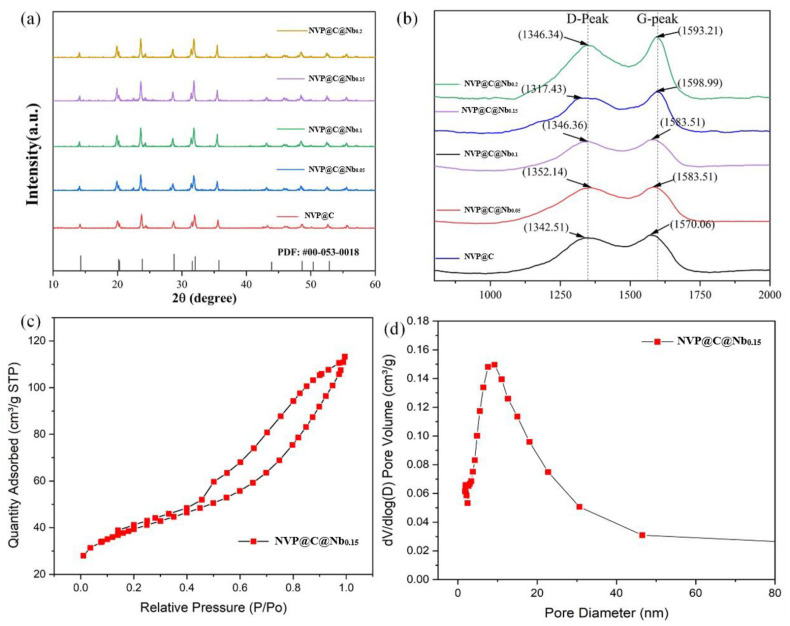
(**a**) XRD patterns of all-sample. (**b**)Raman spectra of all-sample. (**c**) Nitrogen adsorption/desorption isotherms of all samples. (**d**) the pore-size distribution of the NVP@C@Nb_0.15_.

Following the method described above, different compositions of Nb-doped NVP were prepared to optimize their characteristics. The corresponding XRD profiles are given in Figure 2a. The primary peak of the materials was consistent with the PDF card (CPDS-NO.053-0018), indicating that they belonged to the hexagonal system with the R-3c space group [36,37]. The XRD peak shape of NVP@C@Nbx was sharp and well-defined without any other peaks, which indicated that the material had good crystallinity. This suggested that the doping with Nb did not alter the crystal structure of the material. Additionally, a slight shift of the peaks towards a smaller angle was observed, which was due to the fact that the ion radius of Nb^5+^ is slightly bigger than V^3+^ [38]. As shown in Table 1, the position of each peak of XRD was offset to a small angle relative to the PDF card. Consequently, the replacement of V^3+^ by Nb^5+^ increased the interplanar spacing within the NVP framework, which in turn facilitates the intercalation and deintercalation of sodium ions. Raman spectroscopy (Figure 2b) was employed to identify the presence of carbon in the materials. Spectroscopy data of both NVP@C and NVP@C@Nb_0.15_ cathodes exhibited D and G bands, around 1350 and 1595 cm^−1^, respectively, which were typical of carbon with dominants disorder sp2 Raman feature [39]. The peak intensity ratio of the D band to the G band (ID/IG) for the NVP@C and NVP@C@Nb_0.15_ was calculated to be 0.855 and 0.8239, respectively, indicating that both materials had a high graphitization degree and high electrical conductivity. The ID/IG ratio, a measure of the D peak to the G peak, serves as an indicator of the sample material’s level of disorder and graphitization. A decreased ID/IG ratio suggests a higher organized graphite or graphene structures in comparison to the prevalence of defects. The specific surface area and pore size distribution were identified by BET, and the corresponding isotherms were presented in Figure 2c,d. Mesoporous analyses are usually performed using the Barrett–Joiner–Halenda model, which is an application of Kelvin’s equation to the cylinder model for the mesoporous range. The monolayer saturated adsorption volume data, Vm, obtained after treating a small section of data with the BET equation for data segments between P/P_0_ = 0.05 and 0.35, was then used to calculate the specific surface area data. The pore distribution curves revealed that NVP@C@Nb_0.15_ had a specific surface area of 135.07 m^2^/g. This favorable surface area, along with the presence of micro/mesopores, provided abundant active sites and transport channels for Na^+^. This facilitated the utilization of the electrode materials and enhanced the rate of Na^+^ migration. Furthermore, the carbon coating effectively reduced the contact resistance and minimized side reactions between the active material and the electrolyte. This protective layer was benefit to prevent the dissolution of V^3+^, which could otherwise cause the structural collapse of the electrode material. By maintaining the structural integrity of the electrodes, the carbon coating contributed to the overall stability and longevity of SIBs.

**Figure 3:**
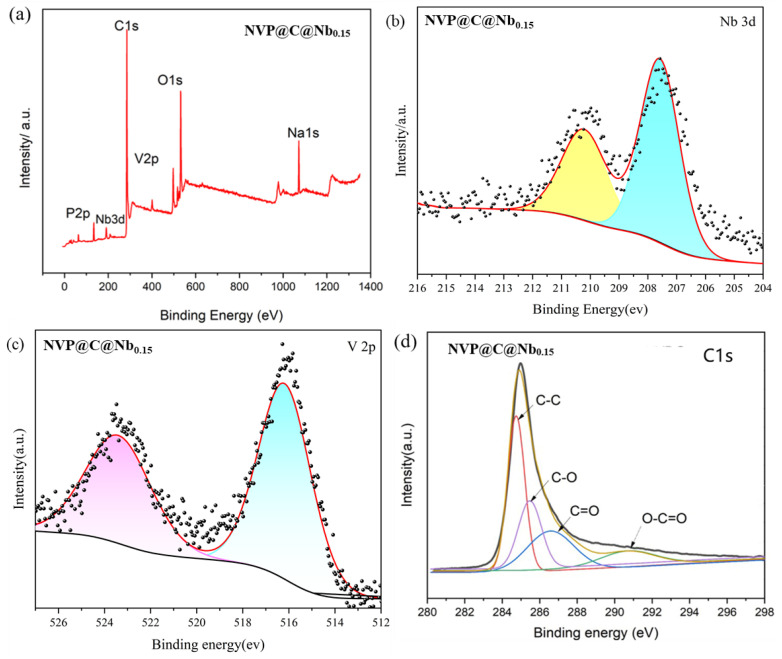
(**a**) Full XPS spectra of NVP@C@Nb_0.15_. (**b**–**d**) High-resolution XPS spectra of Nb 3d, V2p, and C1s of NVP@C@Nb_0.15_.

The valence states of the materials were investigated by XPS, and the results are presented in Figure 3. The survey spectrum for both NVP@C and NVP@C@Nb revealed the presence of elements such as Na, O, V, P, C, and Nb. Specifically, the high-resolution V 2p (Figure 3c) spectrum of NVP@C and NVP@C@Nb could be fitted into two diverse peaks around 517.0 and 524.3 eV, which were assigned to V _2_p^3/2^ and V_2_p^1/2^ orbitals of the V _2_p electronic state, respectively [40]. Nb^5+^ doping can increase the electronic conductivity during the electrochemical reaction and accelerate the embedding and dislodging of Na^+^, which can be seen in V 2p3/2 and V 2p1/2 of V^3+^ in the split-peak spectra of V, indicating that the substitution of Nb for vanadium has not caused any change in the valence state of V. The high-resolution spectrum of Nb 3d orbitals was displayed in Figure 3b; the peaks at 211.25 eV and 208.12 eV could be assigned to Nb 3d^3/2^ and Nb 3d^5/2^ orbitals, which indicated that the Nb still belonged to the ortho-pentavalent state. The high density of the electron cloud of the outer layer of the metal of Nb5^+^ is prone to the formation of the Nb-O bond, and the electronic conductivity can be improved during the process of electrochemical reaction. In the C 1s spectrum (Figure 3d), the four peaks at 285.1, 285.7, 286.9, and 291.9 eV could be attributed to different carbon species: C–C, C–O, C=O, and O–C=O, respectively. These peaks reflected the presence of carbon in various chemical environments within the NVP@C and NVP@C@Nb, which was consistent with the carbon coating and the carbon components of the vanadium phosphate framework.

Overall, the XPS analysis provided insight into the valence states and chemical environments of the elements within the materials, confirming the presence of the desired elements and the effects of Nb doping on the vanadium phosphate structure.

In order to further clarify the mechanism behind the enhanced performance of the cathode material after the incorporation of NVP@C@Nb, density functional theory (DFT) calculations were conducted. The present calculations had been performed using VASP code, which was a powerful computational tool known for its implementation of the PAW method. In this particular study, GGA within the PBE exchange–correlation function was chosen. This function was widely used due to its generally good accuracy for a variety of materials. A plane-wave basis set with a cutoff energy of 400 eV was employed to ensure that the calculations reached a sufficient level of accuracy, with the total energy converging to 1 meV/atom. BZ sampling was carried out using a 4 × 4 × 2 k-points mesh for the bulk crystal. This k-point mesh was chosen to provide an adequate representation of the BZ and to ensure that the calculated electronic properties were converged. During the optimization process, all atoms in the structure were allowed to relax until the maximum absolute forces on all atoms converged to 0.05 eV/Å. This convergence criterion indicated that the atomic positions had been optimized to a level of precision that was considered sufficient for most computational studies.

**Figure 4:**
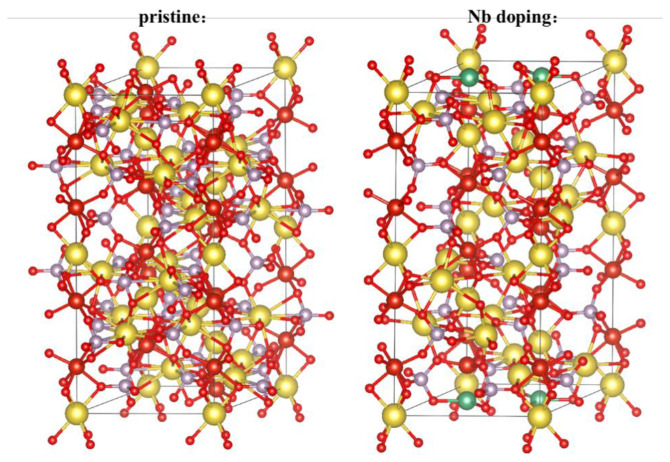
Crystal structure of NVP and NVP@C@Nb_0.15_.

By performing DFT calculations, we could gain insights into the electronic structure, bonding characteristics, and potential energy surfaces of the NVP@C@Nb material. The NVP unit cell consisted of six NVP structural motifs, each containing octahedral VO_6_ and tetrahedral PO_4_ groups. These groups were connected at their corners to form a polyanionomer, [V_2_(PO_4_)_3_], and were further connected along the c-axis direction by PO4 groups. The NVPs have two types of Na^+^ positions, which are referred to as Na1(6b) and Na2(18e) sites (Figure 4):

1. Na1(6b) Site: One of the Na^+^ ions was located at the six-coordinated Na1(6b) site. This site is typically involved in the redox reactions and was responsible for the charge storage capacity of the material;
2. Na2(18e) Site: The other two Na^+^ ions belonged to the same eight-coordinated Na2(18e) site. The Na2(18e) site was associated with the structural framework and was more directly involved in the redox reactions [41,42].

The Nb doping into the NVP structure could alter these properties by introducing additional sites for sodium ion insertion and improving the structural stability, which could lead to enhanced electrochemical performance. The changes in the electronic structure, as indicated by the CV and galvanostatic cycling results, suggested that Nb doping was a useful strategy for improving the properties of NVP as a cathode material in SIBs.

**Figure 5:**
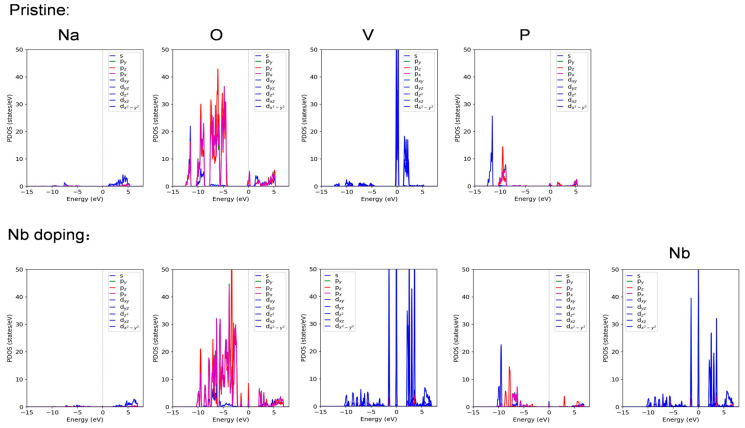
Partial wave density of state.

The results of the DFT calculations indicated that there was a total of 12 vanadium atoms in the unit cell of the system. According to the specified impurity ratio, 15% of these V atoms were replaced by Nb atoms, as shown in the Figure (Figure 4). Analysis of the partial wave density of states (PDOS) revealed that the electrons near the Fermi surface of the system were primarily contributed by the d orbitals of the vanadium atoms (Figure 5). After Nb doping, the d orbitals of the Nb atoms also contributed electrons that are concentrated near the Fermi surface. It could be concluded that the electronic properties of the system, which are crucial for its performance as an electrode material, were predominantly determined by Nb atoms presented in the structure.

The fact that Nb doping had a significant impact on the electronic structure of the system indicated that the introduction of Nb atoms was beneficial for studying and optimizing the material’s properties. Nb, a transition metal, could alter the electronic band structure, improve the electrical conductivity, and possibly enhance the stability and redox reaction kinetics of the material. These findings validated the experimental efforts to dope the vanadium phosphate material with Nb and provided a theoretical basis for further material development and performance optimization.

**Figure 6:**
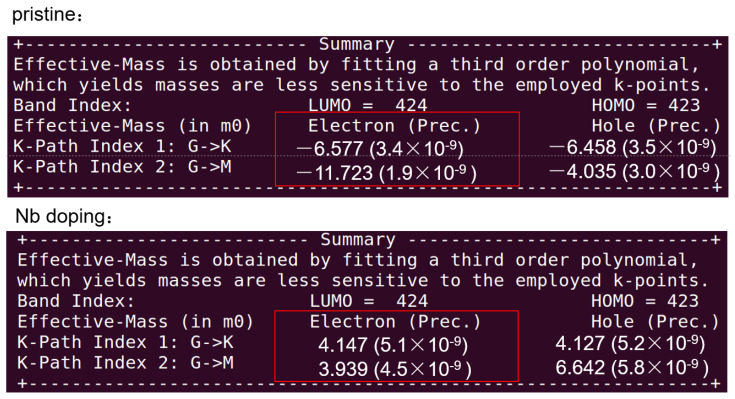
The effective mass of NVP and NVP@C@Nb_0.15_.

The figure depicting the effective mass of the system demonstrated that Nb doping would lead to a decrease in the effective mass in all directions, especially in the direction of the gap minimum (GM) (Figure 6). In a metallic system, the distinction between electrons and holes was not strict, and electrons were typically the default carriers. Therefore, when discussing the effective mass, we focused on the absolute value of the effective mass of electrons. As we all know, a smaller effective mass indicates that electrons experienced less resistance when moving through the material to improve electrical conductivity. The decrease in effective mass after Nb doping suggested that the electrons could move more freely through the material, which is indicative of enhanced electronic mobility.

Higher electronic mobility could lead to better electrical conductivity, which is a critical property for electrode materials in applications such as batteries and solar cells. Therefore, based on these calculation results, it can be concluded that the electrical properties of the system were significantly improved due to Nb doping. This improvement in electrical conductivity could be a key factor contributing to the enhanced performance of the cathode material after the incorporation of NVP@C@Nb.

**Figure 7:**
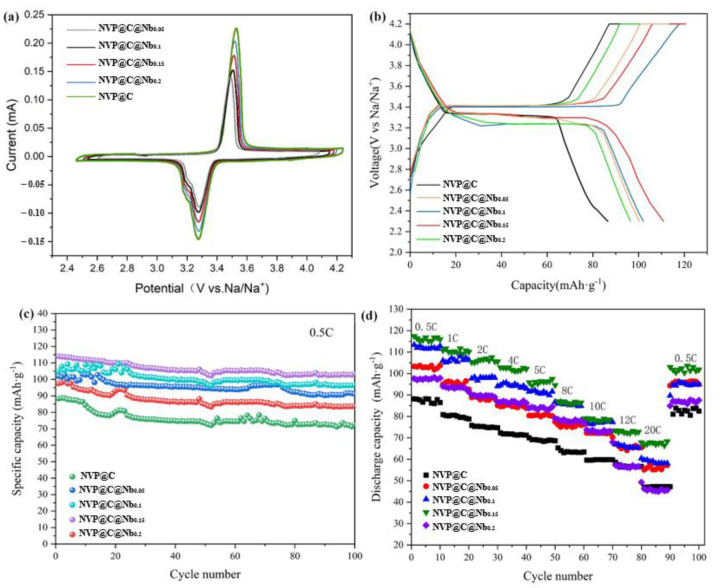
Electrochemical performances of NVP@C and NVP@C@Nb_x_: (**a**) CV patterns at a scan rate of 0.1 mVs^−1^ (**b**) first discharge/charge curves of NVP@C and NVP@C@Nb_x_ (**c**) cycling performance of NVP@C and NVP@C-@Nb_x_ at a current density of 0.5C (**d**) the rate performances of NVP@C and NVP@C@Nb_0.15_.

To evaluate the electrochemical oxidation/reduction features of both cathodes of NVP@C and NVP@C@Nb, cyclic voltammetry (CV) was performed initially (Figure 7a). The CV measurements were conducted in a coin-type R2016 cell configuration with Na metal as the counter electrode and tested in a potential region of 2.5–4.2 V (vs. Na/Na^+^) with 0.1 mV·s^−1^ sweep rate.

For the NVP@C cathode, a typical anodic/cathodic pair of peaks was observed at 3.70/3.12 V during the charging-discharging process. These peaks are associated with the extraction and insertion of Na^+^ ions from the sodium tolerance position in the tetrahedral position of the octal coordination environment, leading to a change in the valence state of V from trivalent to tetravalent. This process maintained the charge neutrality and resulted in a potential plateau of 3.4 V versus Na^+^/Na. The Nb doping did not alter the redox reaction of NVP, as observed in the CV curve. However, after, the oxidation peak in the CV curve shifted to higher potentials, while the reduction peak shifted to lower potentials. These suggested that the redox reaction of the material was enhanced by Nb doping. The near-overlapping peaks in CV curves indicated excellent electrode reversibility and stability for the NVP@C.

The galvanostatic cycling technique was performed to examine the effect of varying amounts of Nb doping on the electrochemical performance of the NVP@C material, which involved applying a constant current to the material and measuring its charging and discharging behavior over multiple cycles. The results are given in Figure 7a. The first charging voltage profiles of NVP@C@Nb materials revealed distinct plateaus at 3.4 V, as shown in Figure 7b. The platform voltages corresponded to the redox reactions of V^3+^ impulse V^2+^. As a result, the charge/discharge profiles of NVP@C displayed a capacity of 97.96/86.5 mAh g^−1^ during the first cycle with an initial coulombic efficiency of 88.3% at the 0.5C rate. The Nb substitution had improved the charge and discharge capacity of NVP@C at 0.5C-rate (NVP@C@Nb_0.15_: 120.6 mAh g^−1^/113.9 mAh g^−1^) with a significantly improved initial coulombic efficiency of 94.44%. This dramatic increase in capacity and efficiency was a strong indication that the electrochemical performance of the material had been significantly enhanced by the Nb doping. The discharge pressure plateau difference for NVP@C@Nb_0.1_ and NVP@C@Nb_0.2_ was smaller than that of NVP, which showed that the polarization of the material became smaller. The reduction in lattice volume caused by Nb doping was believed to shorten the transport channel for sodium ions, which indirectly promotes their transfer. This improvement in ion transport properties contributes to the enhanced electrochemical performance of the NVP@C@Nb materials, making them promising candidates for use in SIBs and other electrochemical energy storage systems. However, it was important to control the amount of niobium doping to avoid excessive polarization and poor cycle stability.

**Figure 8:**
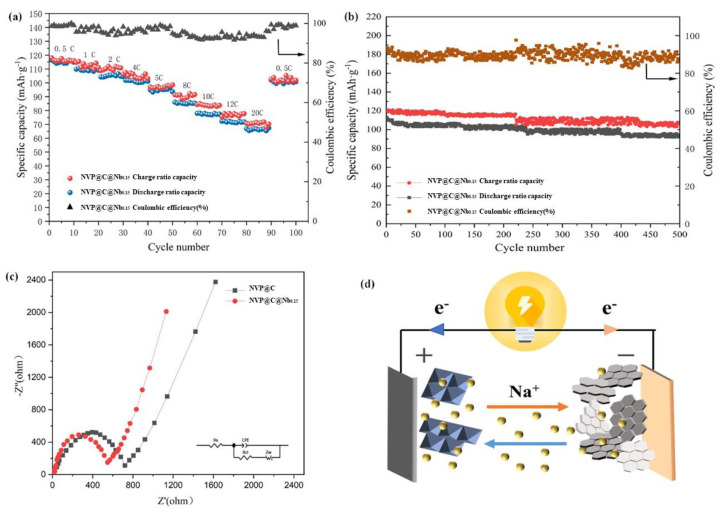
Electrochemical performances of NVP@C and NVP@C@Nb_0.15_: (**a**) the rate performances of NVP@C and NVP@C@Nb_0.15_. (**b**) Long-term cycle performance of NVPF@C@Nb_0.15_ at 0.5C. (**c**) EIS plots of NVP@C and NVP@C@Nb_0.15_ (**d**) Working principle of SIBs.

The cycling performances of the NVP@C and NVP@C@Nb_x_ cathodes were initially evaluated under a low current rate of 0.5C during 100 cycles (Figure 7c). The NVP@C cathode exhibited a lower reversible capacity of 72.5 mAhg^−1^ after 100 cycles. However, the NVP@C@Nb_0.15_ cathodes showed excellent reversible capacity, with a discharge capacity of 104.7 mAhg^−1^ after 100 cycles and a cycling retention of 91.62%. After 500 cycles, the discharge capacity was still 94.5 mAhg^−1^ with a capacity loss rate of only 16.3% (Figure 8b), indicating good long-term stability. Ge’s team [43] had synthesized the carbon-coated monoclinic NaVPO_4_F cathode of SIBs, which reversible capacity was 111 mA hg^−1^ at 0.1C for 1000 cycles at 10C, maintaining 81.8% capacity and 68 mAhg^−1^ at 20C. These showed that the doped heteroatoms could improve the performance of NVP@C.

In order to further explore the effects of Nb doping on battery capacity, the capacities of the NVP@C@Nb_0.15_ cathode were tested over a range of current rates from 0.5C to 20C (Figure 7a). The NVP@C@Nb_0.15_ cathode displayed an excellent rate performance with capacities of 114.2 mAhg^−1^, 107.5 mAhg^−1^, 104.6 mAhg^−1^, 101.72 mAhg^−1^, 100.07 mAhg^−1^, 95.29 mAhg^−1^, 84.94 mAhg^−1^, 78.47 mAhg^−1^, and 71.42 mAhg^−1^ at 0.5C–20C, respectively. This is significantly better than the NVP@C cathode, which only had a capacity of 35 mAhg^−1^ at 20C. When the current rate was returned to 0.5C, the capacity of NVP@C@Nb_0.15_ recovered to 100.32 mAhg^−1^, which was only slightly lower than the initial 0.5C capacity. We compared the capacity and stability of currently existing battery cathodes with the present work, as shown in Table 2. Huang et al. [44] studied the effect of Ti^4+^ doping on active materials and synthesized a series of Na_3_V_2_(PO_4_)_3_-Ti_x_/C (0 ≤ x ≤ 0.2) samples by a one-step solid-state method. The rate performance of all modified samples was better than that of blank samples Na_3_V_2_(PO_4_)_3_-Ti_0.15_/C samples. The maximum discharge-specific capacity is 70.8 mAhg^−1^ at 2C. The results showed that doped heteroatoms could improve the electrochemical reversibility and specific capacity of NVP@C.

The superior electrochemical performance of NVP@C@Nb_0.15_ could be attributed to several factors:

1. NVP@C@Nb_0.15_ has abundant pores and channels, a high specific surface area, and more reactive centers, which shorten the ion diffusion distance and improve the utilization rate of electrode materials;
2. The doping of Nb could improve the conductivity of ions/electrons and achieve rapid charge transfer;
3. Vacancies could increase the number of reactive centers and allow for rapid electron/ion diffusion rates;
4. The carbon layer coating limits crystal growth and forms smaller-sized materials, enhancing the electronic conductivity of the cathode materials and resulting in excellent cycle stability and rate performance.

To understand the dynamic behavior of NVP@C and NVP@C@Nb_0.15_, EIS measurements confirmed the enhanced electrical conductivity and electrochemical reaction kinetics of the NVP@C@Nb_0.15_ electrodes, with a lower charge transfer resistance (540.3 Ω) and higher Na^+^ diffusion coefficients. Both plots consist of a semicircle in a high-to-medium frequency region and a straight line in the low-frequency region (Figure 8c). These findings demonstrated that the substitution of Nb and the carbon-coated were beneficial for improving the electrochemical performance of NVP@C cathode materials.

## 4. Conclusions

The synthesis of NVP@C@Nb_0.15_ cathodes via a sol–gel process followed by high-temperature sintering resulted in a material with a larger specific surface area, a fluffy and porous structure, and improved electrochemical properties. This approach effectively created a microstructure that enhanced the diffusion rate of sodium ions, which was critical for battery performance. The presence of abundant vacancies and a high specific surface area provided enough reactive sites for Na^+^ storage to improve the NVP@C@Nb_0.15_ cathode capacity and cycling stability. The insertion of Nb^5+^ has been shown to be beneficial for expanding the interlayer spacing and the diffusion channel of sodium ions, which indirectly promotes faster ion transport and enhances the cycle stability of the cathodes. The study demonstrated that the Nb substitution and the application of a carbon-coated had a synergistic effect, leading to further improvements in the electrochemical properties of the cathodes. DFT calculations suggested that the d-orbitals of Nb were also concentrated in the Fermi surface, and the effective mass of NVP@C@Nb_0.15_ decreased, electrons were easily migrated, and electronic conductivity was greatly improved. This theoretical insight could guide experimental efforts to optimize the amount of Nb doping and thereby achieve the best balance of properties for high-performance SIB cathodes. The findings suggested that the NVP@C@Nb_0.15_ material held great promise for future development as a high-performance electrode material in SIBs and similar electrochemical energy storage systems.

## Figures and Tables

**Table 1:** The original 2theta range corresponding to each crystal plane.

Crystal Surface	(012)	(104)	(110)	(113)	(024)	(116)	(300)	(208)	(314)	(137)
PDF#053-0018	14.297	20.165	20.305	23.836	28.775	32.054	35.744	43.916	48.651	52.88
NVP@C-Nb_0.05_	14.052	19.911	20.188	23.594	28.601	31.802	35.454	43.108	48.433	52.527
NVP@C-Nb_0.1_	14.114	19.859	20.157	23.594	28.624	31.453	35.485	43.108	48.433	52.496
NVP@C-Nb_0.15_	14.083	19.911	20.189	23.635	28.601	31.843	35.455	43.108	48.434	52.486
NVP@C-Nb_0.2_	14.053	19.860	20.188	23.625	28.621	31.423	35.486	43.108	48.484	52.501
NVP@C-Nb_0.25_	14.032	19.911	20.229	23.635	28.601	31.843	35.4958	43.108	48.516	52.445

**Table 2:** Performance Comparison of Battery Positives.

Material	Composition	Specific Capacity (mAhg^−1^)	Cycling Stability (%)	Rate Capability (mAhg^−1^ at 1C)	Ref.
NVP@C@Nb_0.15_	Na_3_V_2_(PO_4_)_3_@C@Nb_0.15_	114.2 mAhg^−1^, at 0.5C	94.5 (500 cycles)	107.5	This work
NVP@C	Na_3_V_2_(PO_4_)_3_@C	115.1 mAh g^−1^, at 0.1C	91.3 (500 cycles)	99.3	[45]
NVP@C + Fe	Na_3_V_1.85_Fe_0.15_(PO_4_)_3_@C	103.69 mAh g^−1^, at 0.1C	91.45% (1200 cycles)	94.45	[35]
LiFePO_4_	LiFePO_4_	112.4 mAh/g, at 0.1C, 97.1 mAh/g, at 2C	97.8 (100 cycles)	82.5	[46]
NCM (NiCoMn)	LiNi_x_Co_y_Mn_1-x-y_O_2_ (NCM, x ≥ 0.6)	144.0 mAh g^−1^, at 15C	93.4 (200 cycles)	180.3	[47]
NFP	NaFePO_4_	111 mAh/g^−1^, at 0.1C	95 (100 cycles)	52.5	[48]

## Data Availability

The original data presented in the study are included in the article. Additional raw data supporting the conclusions of this article will be made available by the authors on request.
